# Comparative Analysis of Phytochemicals and Antioxidant Characterization Among Different Parts of *Catharanthus roseus*: In Vitro and In Silico Investigation

**DOI:** 10.1155/2024/1904029

**Published:** 2024-10-18

**Authors:** Farjana Akter Hira, Ashekul Islam, Kanika Mitra, Ummey Hafsa Bithi, Khondoker Shahin Ahmed, Sanzida Islam, Shaike Mohammad Abdullah, Md. Nazim Uddin

**Affiliations:** ^1^Department of Biochemistry and Molecular Biology, Mawlana Bhashani Science and Technology University, Tangail 1902, Bangladesh; ^2^Institute of Food Science and Technology, Bangladesh Council of Scientific and Industrial Research (BCSIR), Dhanmodi, Dhaka 1205, Bangladesh

**Keywords:** antioxidants, *Catharanthus roseus*, IC_50_, molecular docking, mPGES-1, phytocompounds

## Abstract

**Background:** The study investigates the antioxidant properties of *Catharanthus roseus*, focusing on identifying its antioxidant compounds and chemical constituents. We compare antioxidant activities across its root, stem, flower, and leaf and examine the inhibition of reactive oxygen species (ROS)–generating enzymes by the plant's phytocompounds.

**Methods:** We conducted a comprehensive analysis that included proximate analysis, mineral content assessment, and in vitro antioxidant characterization of various plant parts—root, stem, flower, and leaf. The levels of bioactive phytochemicals in both ethanol and mixed-solvent extracts of *Catharanthus roseus* were quantified using high-performance liquid chromatography with a diode array detector (HPLC-DAD). Additionally, we performed molecular docking studies to explore the interactions of quantified phytocompounds.

**Results:** HPLC-DAD analysis quantified catechin hydrate, catechol, (−) epicatechin, rutin hydrate, trans-cinnamic acid, quercetin, vanillic acid, kaempferol, and trans-ferulic acid in *Catharanthus roseus.* Despite the ethanol extract having higher total antioxidant properties and flavonoid content, the mixed-solvent extract exhibited higher EC_50_ for reducing power and lower IC_50_ for ABTS, 2,2-diphenyl-1-picrylhydrazyl (DPPH), and metal chelating activities. Molecular docking studies indicated that compounds such as catechin, rutin, epicatechin, quercetin, and kaempferol significantly inhibit the ROS-generating enzyme microsomal prostaglandin E synthase 1 (mPGES-1).

**Conclusions:** The mixed-solvent extract had higher levels of catechin hydrate, rutin hydrate, trans-ferulic acid, and vanillic acid, whereas the ethanol extract contained more (−) epicatechin, catechol, kaempferol, quercetin, and trans-cinnamic acid. While the extracts displayed antioxidant activity, the phytoconstituents also inhibited ROS-generating mPGES-1. These results identify key compounds with potential for developing new chemotherapeutic agents against ROS.

## 1. Introduction

*Catharanthus roseus*, a tropical plant native to Madagascar, is recognized for its significant medicinal properties. *Catharanthus roseus* also named Madagascar periwinkle is a flowering plant member of the family Apocynaceae and is broadly cultured as a pretty plant due to its attractive, long-lasting flowers [1]. It has been introduced to and established in various regions, including India, Pakistan, Malaysia, Bangladesh, and Australia, where it is extensively studied for its therapeutic potential and applications [2–4]. *Catharanthus roseus* is recognized for its medicinal properties and has been traditionally used across different regions to treat a wide range of diseases, including cancer and diabetes. This plant contains 344 bioactive compounds, making it a valuable source of various pharmacological activities, such as anticancer, cytotoxic, antidiabetic, antimicrobial, antioxidant, larvicidal, and pupicidal effects [5]. *Catharanthus roseus* is enriched with alkaloids, flavonoids, carbohydrates, triterpenoids, quinone, tannins, saponins, and phenolic compounds [6]. These bioactive compounds have several functions like antidiabetic, antimicrobial, anthelminthic, antioxidant, antiulcer bactericide, antihypertensive, and anticancer. *Catharanthus roseus* is a significant medicinal plant with abundant sources of amazing health benefits, including alkaloids, and the plant contains bioactive compounds that are used as pharmaceuticals as well as agrochemicals, food additives, and pesticides [7–9]. Different parts of this plant like root, stem, leaf, and flower contain essential chemical constituents. There are many essential phytoconstituents of *Catharanthus roseus* such as vinblastine, vindoliscine, vincristine, vindensine, ajmalicine, reserpine, vincoline, leurosidine, catharanthamine, vincardine, and tabersonine [10]. Ajmalicine is a significant compound that is utilized as an antihypertensive, and another compound named serpentine is used against neuro-inflammatory drugs. Vindolicine is utilized for the production of antidiabetic medicines, while yohimbine, a product of *Catharanthus roseus*, is primarily used in erectile dysfunction treatments [11, 12]. The alkaloids protect against microbial infection and have a broad impact on clinical medicine due to treating hypertension. This plant contains two signature compounds named vincristine and vinblastine which are constituents of drugs used to treat cancer [12, 13].

*Catharanthus roseus* has emerged as a prominent research focus due to its extensive pharmacological activities, antioxidant activities, and its historical use in treating life-threatening diseases. This plant contains flavonoids that have been found to exhibit anti-inflammatory, antioxidant, and anticancer properties. By mitigating inflammation and oxidative stress, these flavonoids may assist in managing blood sugar levels and lowering the risk of complications associated with diabetes [14]. The presence of these phytochemicals imparts significant beneficial activities, including potent antioxidant effects. As a result, *Catharanthus roseus* emerges as a promising alternative for managing and treating various complications, particularly by reducing the production of reactive oxygen species (ROS) [3]. The extract of *Catharanthus roseus* demonstrates powerful antioxidant properties that are vital for neutralizing free radicals and mitigating oxidative stress. These properties play a crucial role in protecting cells from oxidative damage and maintaining overall cellular health [15]. The phytochemicals in *Catharanthus roseus* extracts displayed the most effective free radical scavenging ability and reducing power activity [16]. Alkaloids from *Catharanthus roseus* promoted relatively high glucose uptake in *β*-TC6 and C2C12 cells. At lower concentrations, these alkaloids demonstrated notable antioxidant potential by mitigating H_2_O_2_-induced oxidative damage in *β*-TC6 cells [12]. Researchers can bridge the gap between conventional knowledge and modern science by analyzing its traditional applications and confirming its medicinal potential. Natural antioxidants found in medicinal plants have long been utilized to treat a wide range of illnesses. The claim that *Catharanthus roseus* has a strong antioxidant capacity is supported by all of the pharmacological activities of the plant that have previously been reported.

Our study aimed to explore the compounds responsible for antioxidant activity and to characterize the phytochemical composition in different parts of *Catharanthus roseus*. To meet these objectives, we conducted a comparative analysis of antioxidant activities across the plant's root, stem, flower, and leaf. Additionally, our study aimed to delve into the antioxidant properties of compounds from *Catharanthus roseus* by employing molecular docking techniques. This involved assessing the interaction between the compounds and a ROS-generating enzyme, providing insights into the potential therapeutic applications of these compounds in combating oxidative stress.

## 2. Materials and Methods

Chemicals and reagents: Merck India supplied the drugs DPPH and ABTS. We bought methanol, hydrochloric acid, sodium hydroxide, aluminum chloride, sodium carbonate, potassium ferricyanide, and other chemicals from Merck in Darmstadt, Germany. The Folin–Ciocalteu reagent, ascorbic acid, tannic acid, catechin, and ferric chloride were all acquired from Sigma Co. (St. Louis, Missouri, USA). The companies BDH Co. and Loba, India, provided the ascorbic acid, NBT, and ferrozine. Analytical-grade substances make up the chemicals and reagents.

For the HPLC-DAD method, gallic acid, 3,4-dihydroxybenzoic acid, catechin hydrate, catechol, (−) epicatechin, caffeic acid, vanillic acid, syringic acid, rutin hydrate, p-coumaric acid, *trans*-ferulic acid, rosmarinic acid, myricetin, quercetin, trans-Cinnamic acid, and kaempferol were purchased from Sigma-Aldrich (St. Louis, MO, USA). Acetonitrile (HPLC), methanol (HPLC), acetic acid (HPLC), and ethanol were obtained from Merck (Darmstadt, Germany).

### 2.1. Collection and Sample Preparation

We collected *Catharanthus roseus* from the IFST, BCSIR laboratory garden, in October 2022. After collection, the plant parts were sorted into root, stem, flower, and leaves. To ensure high grading quality, the sample was washed with fresh water. Then the different parts of the plant were separately cut down into smaller pieces and prepared for air drying along with oven drying. The powder form of the sample was produced by grinding the dried plant parts using a grinder and storing them in a desiccator to avoid moisture.

### 2.2. Preparation of Sample Extracts

Two types of extracts were prepared in the extraction procedure; they were ethanol solvent and mixed solvent (methanol, chloroform, and water). Ethanol extract from four samples was prepared by dissolving them in ethanol [17]. 25 g of each sample was dissolved in 225 mL ethanol in flat-bottomed containers. Then with occasional shaking and stirring, good mixing was confirmed, and finally it was subjected to sonication for 30 min. This process was repeated five times to collect ethanol extract from every specific sample. By filtering the mixture using Whatman No. 1 filter paper, supernatant ethanol extract was obtained. Then the sample was made ready by evaporating the solvent. Mixed-solvent extracts of the samples were prepared by using 60% methanol, 25% chloroform, and 15% water [18, 19]. 50 g of each sample in flat-bottom containers was dissolved in 450 mL mixed solvent. In a similar way to the ethanol extract collection procedure, further mixed-solvent extract collection proceeded to get the final extract.

### 2.3. Proximate Analysis

Following the procedure recommended by the Association of Official Analytical Chemists (AOAC), the amount of moisture, ash, protein, fat, and crude fiber was measured. To calculate the moisture content of samples, evaporation weight loss at 105°C for 6–8 h was used [20]. Considering ash content measurement, a muffle furnace that burned at 600°C for 6 h to produce white ash was used to measure the amount of ash present [21]. The Kjeldahl method is a widely used technique for calculating protein content, which had three steps: digestion, distillation, and titration [22]. This method was used to determine the protein content of samples by multiplying the nitrogen with 6.25 to convert it into protein. In the fat determination process, by extracting them in hexane and measuring the residues left behind after the hexane has evaporated, the amount of fat in each sample was measured. To determine the crude fiber content, fat-free samples were taken, boiled in 200 mL (1.25%) sulfuric acid over reflux, filtered, and then washed with hot water to remove any acidity. To make the sample non-alkaline, the residue was then boiled once more with 200 mL (1.25%) NaOH before filtration and washing with hot water. After cooling, it was weighed. We incinerated the crude fiber in a muffle furnace at roughly 600°C for 20 min, cooled it, weighed it, and estimated the amount of fiber produced. By deducting the sum of ash, protein, fat, moisture, and crude fiber content from 100, the amount of carbohydrates was calculated. The energy was calculated using energy conversion factors, also referred to as Atwater factors [21, 23].

### 2.4. Mineral Determination

Following the procedures outlined in the AOAC Manual of Laboratory Techniques, minerals (sodium, potassium, calcium, iron, zinc, manganese, and magnesium) were examined. Ash samples were burnt in a muffle furnace for 6 h at 600°C. The stock solution was made by mixing burnt samples with 6 M HCL, and the mineral content was assessed using an atomic absorption spectrophotometer (AAS; Thermo Scientific, ICE 3000 series, USA) [24].

### 2.5. Determination of Phytochemical Profile

The phytochemical composition of the extracts was examined using HPLC. HPLC is a crucial qualitative and quantitative method frequently employed for pharmaceutical and biological sample quantification.

#### 2.5.1. Preparation of Working Standard Solutions

Stock standard solutions were made by dissolving 16 phenolic compounds in methanol in a 25 mL volumetric flask. The range of stock solutions' concentrations was 4.0–50 g/mL. The right amounts of each stock solution were combined, and the working standard solutions were created by serial dilution. Every solution was kept cold for storage.

#### 2.5.2. HPLC Analysis

Detection and quantification of selected polyphenolic compounds in ethanol and mixed-solvent extracts were determined by HPLC-DAD analysis as described by Uddin et al. [25, 26] with some modifications. HPLC analysis was conducted using a Shimadzu LC-20A system from Japan, featuring a binary solvent delivery pump (LC-20AT), a column oven (CTO-20A), an autosampler (SIL-20A HT), and a photodiode array detector (SPD-M20A), all managed by Lab Solution software. The separation utilized a Luna C18 (5μm) Phenomenex column (4.6 × 250 mm) at a temperature of 33°C. The mobile phase composed of A (1% acetic acid in acetonitrile) and B (1% acetic acid in water) with gradient elution: 0.01–20 min (5%–25% A), 20–30 min (25%–40% A), 30–35 min (40%–60% A), 35–40 min (60%–30% A), 40–45 min (30%–5% A), and 45–50 min (5% A), was used in this study. 20 L of sample was injected, and the flow rate was set at 0.5 mL/min. To validate the procedure and analysis, the UV detector was applied with a setting of 270 nm. The mobile phase was degassed under vacuum after being filtered using a 0.45 m Nylon 6, 6 membrane filter. To create the calibration curve, a standard stock solution in methanol containing the following ingredients was created: gallic acid (20 g/mL); 3,4-dihydroxybenzoic acid (15 g/mL); catechin hydrate (50 g/mL); catechol, (−) epicatechin, and rosmarinic acid (30 g/mL each); caffeic acid, vanillic acid, syringic acid, rutin hydrate, p-coumaric acid, *trans*-ferulic acid, and quercetin (10 *μ*g/mL each); myricetin and kaempferol (8 *μ*g/mL each); and trans-cinnamic acid (4 *μ*g/mL) [18, 19].

### 2.6. Determination of Total Phenolic Contents (TPCs)

The amount of TPC was determined following the established method with some modifications by using the Folin–Ciocalteu reagent and quantified as gallic acid equivalents. Each of the four samples was mixed with 0.5 ml of Folin–Ciocalteu reagent (0.5 N) using 0.5 ml of extract at a concentration of 1.0 mg/ml, and the mixtures were incubated at room temperature for 5 minutes [24]. Then 2.0 mL saturated sodium carbonate was added. Afterward, these were incubated for 30 min at room temperature and absorbance was measured at 765 nm. Gallic acid was used as a positive control. The contents of TPC were extrapolated by using the linear equation of gallic acid.

### 2.7. Determination of Total Flavonoid Contents

In each sample, individually 1 mL of test sample and 4 mL of water were added to a volumetric flask. After adding 0.3 mL of 5% sodium nitrite, we have to wait 5 minutes before adding 0.3 mL of 10% aluminum chloride. After the mixture had been incubated for 6 min at room temperature, 2 mL of 1 M sodium hydroxide was added to the reaction mixture. Immediately, distilled water was used to bring the final volume to 10 mL. Spectrophotometrically, the absorbance of the reaction mixture was measured at 510 nm in comparison to a blank. Total flavonoid content was calculated as (mcg/100 g) using the equation based on the calibration curve. Rutin hydrate was used as a reference to determine the total flavonoid concentration.

### 2.8. Determination of Total Antioxidant Capacity

Total antioxidant activity was determined by using the phosphomolybdenum blue and ferric reducing antioxidant power (FRAP) assay. The determination of total antioxidant activity was done using the phosphomolybdenum method with slight modifications [27]. The basic principle is based on the reduction of Mo (VI) to Mo (V) by the extract and subsequent formation of a green phosphate Mo (V) complex at acidic pH. 0.3–0.5 mL extract of each sample was combined individually with a mixture of 3 mL of reagent solution (0.6 M sulfuric acid, 28 mM sodium phosphate, and 4 mM ammonium molybdate). At 95°C, the tubes containing the reaction solution were then capped and incubated for 90 min. After the samples had cooled to room temperature, the absorbance of the solution was then measured at 695 nm against blank. The antioxidant activity is expressed as the mcg of the equivalent of gallic acid for *Catharanthus roseus.*

Furthermore, total antioxidant was also determined by FRAP assay. FRAP assay was carried out according to the method with some modifications. FRAP reagent was prepared from acetate buffer (0.31 g sodium acetate and 1.6 mL acetic acid make up to 100 mL) (pH 3.6), 10 mM TPTZ solution in 40 mM HCL, and 20 mM iron (III) chloride solution in the proportion of 10:1:1 (v/v), respectively. The FRAP reagent was freshly produced each day and reheated to 37°C in an oven before use. 0.5 mL of sample extract and 0.5 mL of water were combined with 3 mL of FRAP reagent and the solvents were thoroughly mixed. The absorbance was measured at 593 nm using a UV-visible spectrophotometer (UV-VIS 1200, Shimadzu Corporation, and Japan). A standard curve of ascorbic acid was prepared by applying a similar procedure.

### 2.9. Determination of ABTS Scavenging Capacity

The ABTS radical cation decolorization method, which was previously reported, was used to measure the free radical scavenging activity. ABTS was prepared at a concentration of 7 mmol/L (0.03841 g dissolved in 10 mL), and potassium peroxodisulfate was prepared at 4.95 mmol/L (0.01338 g in 10 mL). Both were mixed and dissolved in ACS-grade water. The solution was then diluted with distilled water in a 1:9 v/v ratio (10 mL is quantitatively transferred into a 100 mL calibrated flask and diluted). The reagent can be used for 7 days if kept in the dark at 4°C after the solution has been incubated for 12 h in the dark. Different volume samples were pipetted into five test tubes and water was added into tubes for a total volume of 1.0 mL. Then 3 mL ABTS reagent was added and the mixture was incubated for 5 min. Absorbance was measured at 670 nm. Control was prepared with water and reagent.

The percentage of inhibition can be calculated using the following formula:

(1) $$\begin{array}{c} \text{Inhibition}\left(\%\right)=\left(\frac{A_{0}-A_{1}}{A_{0}}\right)\times 100, \end{array}$$

where *A*_0_ is the absorbance of the control and *A*_1_ is the absorbance of the test.

### 2.10. DPPH Inhibition Assay

DPPH is a stable free radical that is frequently used to evaluate the antioxidant compounds' capacity to scavenge radicals. The free radical scavenging activity of four samples was measured by using DPPH method in our previous study [24]. The reaction mixture (3.0 mL) is composed, in brief, of 1.0 mL of varying concentrations of extract and 2.0 mL of DPPH in methanol (0.004%). In a dark environment, the mixture was incubated for 10 min. The absorbance is measured at 517 nm against methanol as a blank and a control is prepared using DPPH and methanol instead of the sample extract. Ascorbic acid serves as the positive control in this experiment. The percentage of inhibition can be calculated using the following formula:

(2) $$\begin{array}{c} \text{Inhibition}\left(\%\right)=\left(\frac{A_{0}-A_{1}}{A_{0}}\right)\times 100, \end{array}$$

where *A*_0_ is the absorbance of the control and *A*_1_ is the absorbance of the test.

Then IC_50_ is determined extrapolating the graph of scavenging activity versus the concentration of extract.

### 2.11. Determination of Metal Chelating Capacity

Chelating agents are organic or inorganic substances with the capacity to bind to harmful metal ions and build intricate structures that can be quickly eliminated from the body. The metal chelating activity was assessed by first adding 0.1 mM FeSO_4_ (0.2 mL) and 0.25 mM ferrozine (0.4 mL) to various concentrations of plant extract. Later, the mixture was incubated for 10 min at room temperature, and the mixture's absorbance at 562 nm was measured.

The following formula was used to determine the amount of cheating activity:

(3) $$\begin{array}{c} \text{Inhibition}\left(\%\right)=\left(\frac{A_{0}-A_{1}}{A_{0}}\right)\times 100, \end{array}$$

where *A*_0_ is the absorbance of the control and *A*_1_ is the absorbance of the test.

Then IC_50_ is determined extrapolating the graph of scavenging activity versus the concentration of extract.

### 2.12. Determination of Reducing Power

The reducing power can be determined by the method described in [28]. Different concentrations of the extracts were combined with 2.5 ml of phosphate buffer and 2.5 ml of potassium ferricyanide solution. For 20 min, this mixture was maintained in a water bath at 50°C. 2.5 mL of 10% trichloroacetic acid was added after cooling and centrifuged for 10 min at 3000 rpm. A freshly made ferric chloride solution (0.5 mL) and distilled water (2.5 mL) were combined with the solution's upper layer (2.5 mL). At 700 nm, the absorbance was measured. Similar to this procedure without the samples, the controls were constructed. Standardization involved the use of ascorbic acid at a range of concentrations. Increasing reducing power is indicated by the reaction mixture's increased absorbance. EC_50_ refers to the concentration of the extract or standard needed to achieve an absorbance of 0.50.

### 2.13. Molecular Docking Study

#### 2.13.1. Selection and Preparation of Protein and Compounds for Conducting Molecular Docking

We specifically selected the ROS-generating microsomal prostaglandin E synthase 1 (mPGES-1) (4YL3) and quantified all phyto compounds using HPLC to investigate their potential relationship with the inhibition of this enzyme. The protein was downloaded from the Research Collaboratory for Structural Bioinformatics Protein Data Bank (RCSB PDB) (https://www.rcsb.org/) and prepared by removing water and hetero-atom by using the BIOVIA Discovery Studio software (https://discover.3ds.com/discovery-studio-visualizer-download). The Simplified Molecular Input Line Entry System (SMILES) structure of all selected compounds was downloaded from PubChem (https://pubchem.ncbi.nlm.nih.gov/) and converted into the structural data file (SDF) format by using DataWarrior software (https://openmolecules.org/datawarrior/).

#### 2.13.2. Molecular Docking

We used PyRx software (https://pyrx.sourceforge.io/) for conducting the molecular docking study. First, we identified the binding pocket of the selected protein by using the PrankWeb tool (https://prankweb.cz/). PrankWeb builds upon a machine learning–based method for the prediction of ligand binding sites from protein structure. We converted the protein into a protein data bank with charges and atom types (PDBQT) format and selected the binding site-associated amino acid residues for making a grid. We minimized the energy of ligands and converted them into PDBQT format for further steps. Finally, we conducted the docking study to identify the docking score between the selected enzyme and ligands.

### 2.14. Statistical Analysis

The experiments were conducted in triplicate, and the data were presented using the mean and standard deviation. For group comparisons, a one-way ANOVA was conducted, followed by Tukey's honestly significant difference (HSD) test for pairwise comparisons between two groups, utilizing GraphPad Prism 8.0. Additionally, a two-way ANOVA was employed to compare groups based on two distinct categorical variables. Statistical significance was defined at ⁣^∗^*p*  <  0.05.

## 3. Results

### 3.1. Proximate Composition of Different Parts of *Catharanthus roseus*

Proximate analysis reports the quantity of protein, ash, fat, moisture, fiber, carbohydrates, and energy content of the sample. The proximate content of each of the four plant sections (root, stem, flower, leaf) was evaluated. The comparative representation of each nutrient amount in terms of mean with standard deviation value is shown in Table 1. The statistical information showed a significant difference among the different parts. The moisture content ranged from 2.87% to 10.14%. It has been found that the flower contained the highest moisture about 10.14% highly significant (⁣^∗∗∗^*p* < 0.001). Ash contents were evaluated within a range of a minimum of 5.50% in the stem to a maximum value of 11.88% in the leaf. The protein content was determined approximately in the range of 13.62% to 5.47%. It has been found that the leaf contained higher protein about 13.62% than other parts of the plant, whereas the stem contains the lowest protein about 5.47%. The result is statistically significant (⁣^∗∗∗^*p* < 0.001). The highest fat value was found at 6.05% in the leaf with the lowest value at 1.23% in the root. The carbohydrate content of the root, stem, flower, and leaf was about 38.08, 26.57, 60.37%, and 54.88%, respectively. The highest value of carbohydrates was 60.37% in flower. It has been found that the stem contains the highest fiber about 54.18%; the root contains lower fiber about 9.68%. The result evaluated that the leaf contains higher energy about 326.54 kcal/100 g (⁣^∗∗∗^*p* < 0.001) than other parts of the plant and the stem contains lower energy about 168.03 kcal/100 g.

### 3.2. Micronutrients in Different Parts of *Catharanthus roseus*

**Figure 1:**
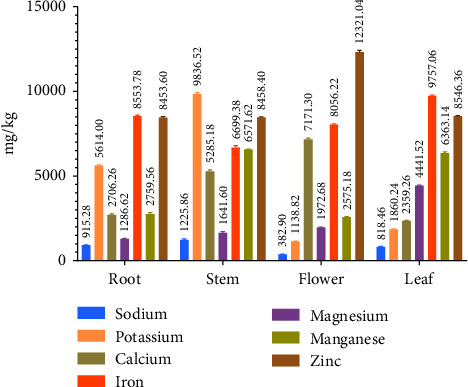
The content of minerals in different parts of *Catharanthus roseus*. The bar indicated the content of sodium, potassium, calcium, iron, magnesium, manganese, and zinc. Data are shown as Mean with standard deviation for triplicate experiments.

Mineral analysis is crucial in the determination of the nutritional values of foods. To establish the micronutrient status of various parts of *Catharanthus roseus*, we examined the mineral contents such as sodium, potassium, calcium, iron, zinc, phosphorus, and magnesium. According to the findings of the current study, *Catharanthus roseus* implies high values for minerals shown in Figure 1 and Table 2 together with a comparison of all analyzed samples and showed significant differences (⁣^∗∗^*p* < 0.01) among the individual minerals values of each sample. For sodium, the stem exhibited the highest concentration at 1225.86 mg/kg, while the flower had a lower concentration of 382 mg/kg. Additionally, the potassium measurement ranged from 1138.82 mg/kg in the flower with the lowest value and the highest value in the stem was 9836.52 mg/kg. Calcium content was higher in the flower (9836.52 mg/kg) and lower in the leaf (2359.26 mg/kg) in comparison to other parts. In the case of magnesium content, it was higher in flower at about 4441.52 mg/kg and lower in root (1286.62 mg/kg). Iron content was higher in the leaf (9757.06 mg/kg) and lower in the stem (6699.38 mg/kg) in comparison to other parts. Manganese content was higher in the stem (6571.62 mg/kg) and lower in the flower (2575.18 mg/kg). Zinc content was higher in the flower (12,321.04 mg/kg) and lower in the root (8453.6 mg/kg).

### 3.3. Phytochemical Profile

**Figure 2:**
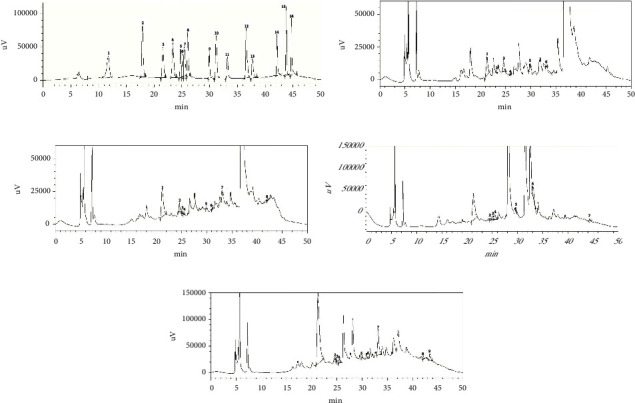
(a) HPLC chromatogram of standard solution ((1) gallic acid, (2) 3,4-dihydroxybenzoic acid, (3) catechin hydrate, (4) catechol, (5) (−) epicatechin, (6) caffeic acid, (7) vanillic acid, (8) syringic acid, (9) rutin hydrate, (10) p-coumaric acid, (11) trans-ferulic acid, (12) rosmarinic acid, (13) myricetin, (14) quercetin, (15) trans-cinnamic acid, and (16) kaempferol). (b) HPLC chromatogram of mixed solvent of root extract ((1) catechin hydrate, (2) catechol, (3) (−) epicatechin, (4) syringic acid, (5) rutin hydrate, and (6) trans-ferulic acid). (c) HPLC chromatogram of mixed solvent of stem extract ((1) catechin hydrate, (2) (−) epicatechin, (3) caffeic acid, (4) vanillic acid, (5) rutin hydrate, (6) p-coumaric acid, (7) trans-ferulic acid, and (8) quercetin). (d) HPLC chromatogram of mixed solvent of flower extract ((1) (−) epicatechin, (2) rutin hydrate, (3) trans-ferulic acid, (4) rosmarinic acid, (5) myricetin, (6) quercetin, and (7) kaempferol). (e) HPLC chromatogram of mixed solvent of leaf extract ((1) catechin hydrate, (2) (−) epicatechin, (3) caffeic acid, (4) vanillic acid, (5) rutin hydrate, (6) p-coumaric acid, (7) trans-ferulic acid, (8) quercetin, and (9) trans-cinnamic acid).

**Figure 3:**
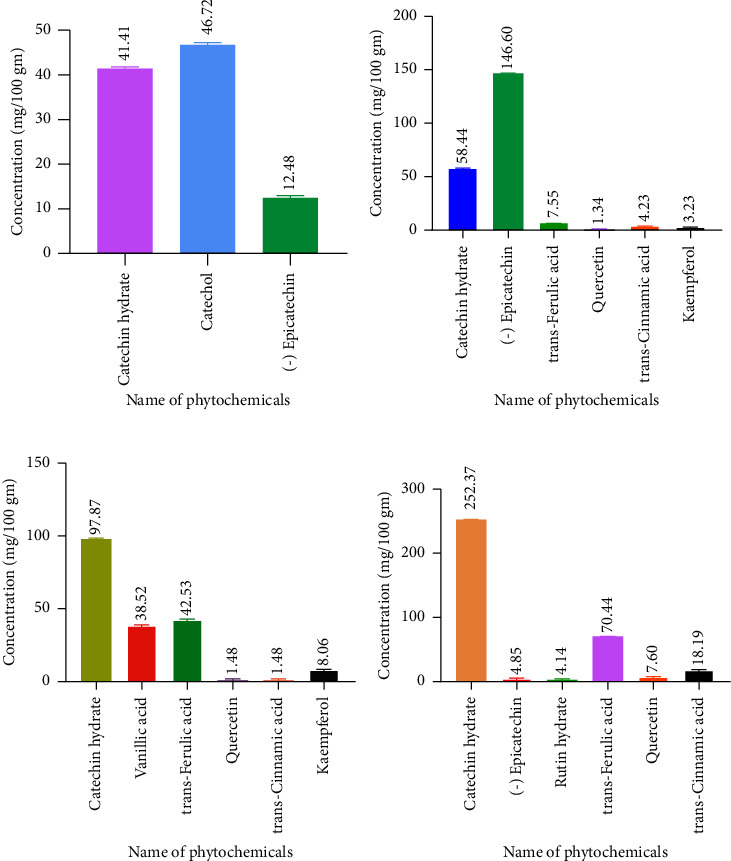
HPLC-based chromatographic quantification of phytochemical in ethanol extracts. (a) Average phytochemical content of ethanol extract in root. (b) Average phytochemical content of ethanol extract of the stem. (c) Average phytochemical content of ethanol extract of flower. (d) Average phytochemical content of ethanol extract of leaf. A one-way ANOVA test was performed and the result showed that the concentration of phytochemicals of different compounds is significantly different (⁣^∗∗∗^*p* < 0.001).

**Figure 4:**
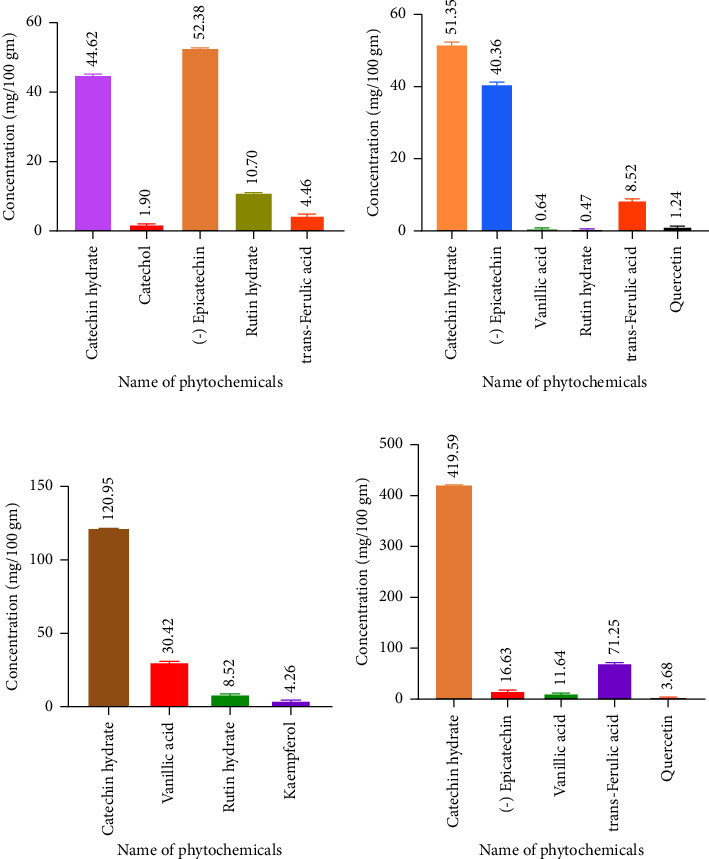
HPLC-based chromatographic quantification of phytochemical in mixed-solvent extracts. (a) Average phytochemical content of mixed-solvent extract in root. (b) Average phytochemical content of mixed-solvent extract of stem. (c) Average phytochemical content of mixed-solvent extract of flower. (d) Average phytochemical content of mixed-solvent extract of leaf. A one-way ANOVA test was performed and the result showed that the concentration of phytochemicals of different compounds is significantly different (⁣^∗∗∗^*p* < 0.0001).

HPLC was used to identify and measure phytochemicals in the sample extracts.  Figure 2 displays the HPLC chromatograms for both the standard solution and the mixed-solvent extracts from the root, stem, flower, and leaf samples. These chromatograms illustrate the separation and identification of various compounds within each sample, providing a detailed profile of their chemical composition. Figures 3 and 4, respectively, demonstrate the chromatographic separations of polyphenols in ethanol extract and mixed-solvent extracts. The outcomes of the experiment revealed that the ethanol extract of *Catharanthus roseus* had a high concentration of catechin hydrate (252.37 mg/100 g of dry extract) which is found in the leaf sample. Catechol (46.72 mg/100 g), (−) epicatechin (146.60 mg/100 g), rutin hydrate (4.14 mg/100 g), and kaempferol (8.06 mg/100 g) were found higher, respectively, in root, stem, leaf, and flower, as shown in Figure 2. Not only RH but also trans-ferulic acid (70.75 mg/100 g), quercetin (7.60 mg/100 g), and trans-cinnamic acid (18.19 mg/100 g) were found in higher amounts in leaf.

The experimental result also showed that the mixed-solvent extract of *Catharanthus roseus* contained an especially high concentration of catechin hydrate (419.59 mg/100 g) which is found in the leaf sample. In addition to catechin hydrate, the leaf was abundant in trans-ferulic acid (71.25 mg/100 g) and quercetin (3.68 mg/100 g). Catechol (1.90 mg/100 g), (−) epicatechin (52.38 mg/100 g), and rutin hydrate (10.70 mg/100 g) were higher in the root. Vanillic acid (30.42 mg/100 g) and kaempferol (4.26 mg/100 g) were higher in flowers in comparison to other parts. All samples experimented were in dry extract.

### 3.4. Comparative Phenolic and Flavonoid Contents

**Figure 5:**
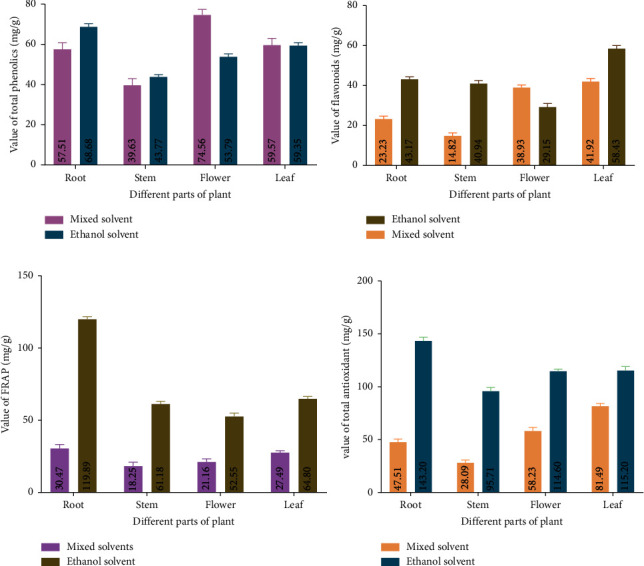
Phytochemicals and antioxidant contents of different solvent extracts of different parts in *Catharanthus roseus*. (a) The bar indicated the content of phenolics in ethanol and mixed-solvent (methanol + chloroform + water) extract, (b) flavonoids in ethanol and mixed solvent (methanol + chloroform + water), (c) antioxidants by FRAP method in ethanol and mixed solvent (methanol + chloroform + water), and (d) total antioxidant by phosphomolybdenum blue method in ethanol and mixed solvent (methanol + chloroform + water). Data are shown as mean with standard deviation for triplicate experiments. We performed two-way ANOVA to compare groups on two different categorical variables.

TPC both in ethanol and methanol + chloroform + water (mixed solvent) extracts is expressed in terms of gallic acid equivalent (the standard equation *y* = 40.095*x* + 0.7235, *R*^2^ = 0.9984).  Figure 5(a) shows that the TPC was about 68.68 mg in the ethanol extract which is higher than 57.51 mg in the mixed-solvent extract of root. It was also found that the total mixed-solvent phenolic content was about 43.76 mg in ethanol extract which was higher than 39.63 mg in mixed-solvent extract of the stem. TPC was about 59.34 mg in the ethanol extract which was lower than 74.56 mg in the mixed-solvent extract of flower. For the leaf, in the case of both extracts, TPC was about 53.79 mg in the ethanol extract and 59.56 mg in the mixed-solvent extract of the leaf. There is a significant difference (⁣^∗∗∗^*p* < 0.001) in the total phenolic content between the ethanol extract and the mixed-solvent extract, as well as notable differences among the extracts from various parts of the plant.

Total flavonoid content both in ethanol and methanol + chloroform + water (mixed solvent) extracts is expressed in terms of rutin hydrate equivalent (the standard equation *y* = 255.28*x* + 8.8426, *R*^2^ = 0.9994). Our findings revealed that the total flavonoid content was about 43.16 mg in ethanol extract which is higher in mixed-solvent extract of root (23.22 mg). It was also found that the total flavonoid content was about 40.93 mg in the ethanol extract which is higher than 14.81 mg in the mixed-solvent extract of stem. Total flavonoid content was about 29.15 mg in the ethanol extract which was lower than 38.93 mg in the mixed-solvent extract of the flower summarized in Figure 5(b). The total flavonoid content was about 58.40 mg in the ethanol extract and 41.92 mg in the mixed-solvent extract of the leaf. A significant difference exists between the total flavonoid content of the ethanol extract and the mixed-solvent extract, with a notable difference (⁣^∗∗∗^*p* < 0.001) among the various extracts from different plant parts, as shown in Figure 5.

### 3.5. Total Antioxidant Contents

Phosphomolybdenum blue techniques were used to measure the total antioxidant content of the root, stem, flower, and leaf against a gallic acid standard. According to the findings, both ethanolic and mixed-solvent extract samples' total antioxidant contents were significantly different from one another (⁣^∗∗∗^*p* < 0.001) (Figure 5(d)). The findings showed that ethanol extract had significantly higher total antioxidant (mg) than mixed-solvent extract in the case of all groups. Total antioxidant content both in ethanol and methanol + chloroform + water (mixed solvent) extracts was expressed in terms of gallic acid equivalent (the standard equation *y* = 72.695*x* − 0.1646, *R*^2^ = 0.9923). The total antioxidant content was about 143.20 mg in the ethanol extract and 47.50 mg in the mixed-solvent extract of root. The total flavonoid content was about 95.71 mg in the ethanol extract which is higher than 28.09 mg in the mixed-solvent extract of the stem. The total antioxidant content was about 115.20 mg in the ethanol extract and 58.22 mg in the mixed-solvent extract of the flower. In the case of the leaf, it was about 132.76 mg in the ethanol extract and 81.48 mg in the mixed-solvent extract.

Similar to this, we used the FRAP method to evaluate the antioxidant levels of several samples of *Catharanthus roseus* extract, with ascorbic acid serving as a standard. According to the findings, the antioxidant content identified by the FRAP assay considerably (⁣^∗∗∗^*p* < 0.001) differs between various solvents (Figure 5(c)). Total FRAP content both in ethanol and methanol + chloroform + water (mixed solvent) extracts is expressed in terms of ascorbic acid equivalent (the standard equation *y* = 22.848*x* + 0.0961, *R*^2^ = 0.9996). FRAP content was about 119.89 ± 1.88 mg in ethanol extract which is higher in mixed-solvent extract (30.47 mg) of root. The result showed that the total FRAP content was higher about 61.17 mg in the ethanol extract of the stem. It was also found that the total FRAP content was about 52.55 mg in the ethanol extract, whereas it was 21.15 mg in the mixed-solvent extract of the flower. Total antioxidant content was about 64.80 ± 1.77 mg in ethanol extract which is higher than 27.49 ± 1.47 mg in mixed-solvent extract of leaf.

### 3.6. Free Radical Scavenging Activities

**Figure 6:**
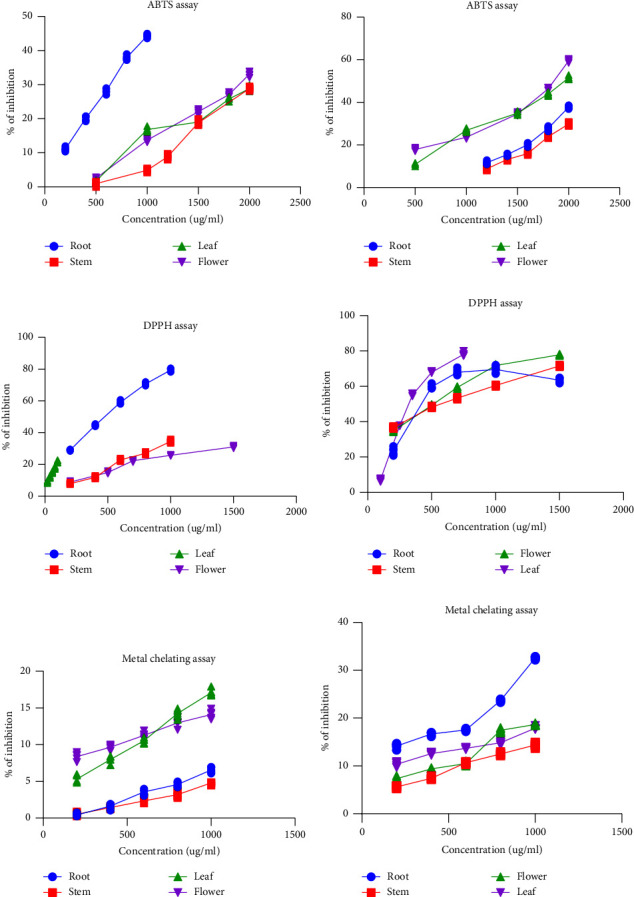
The percentages of inhibition of different extracts in antioxidative assays. (a) The percentages of inhibition of ABTS in ethanol extract. (b) The percentages of inhibition of ABTS in mixed-solvent extract. (c) The percentages of inhibition of DPPH assay in ethanol extract. (d) The percentages of inhibition of DPPH assay in mixed-solvent extract. (e) The percentages of inhibition of metal chelating capacity in ethanol extract. (f) Percentages of inhibition of metal chelating capacity in mixed-solvent extract.

The capacity of the extract to scavenge free radicals in vitro was examined. In this study, many in vitro free radical scavenging models, including ABTS, DPPH, metal chelating activity, and reducing power assay, were used. The results are expressed as the IC_50_ (the minimum concentration needed to reduce or inhibit 50% of free radicals), which is equivalent to the gallic acid standard for the ABTS and DPPH assays and ascorbic acid for the reducing power assay. According to the results, our samples could inhibit free radicals expressed as a percentage of inhibition in Figure 6. There are significant differences between the IC_50_ values for the ABTS assay as well as for the DPPH assay and metal chelating assay in the four solvent extraction groups. The capacity of extracts to scavenge free radicals is indicated by their lower IC_50_ values.

#### 3.6.1. ABTS Scavenging Activity

**Figure 7:**
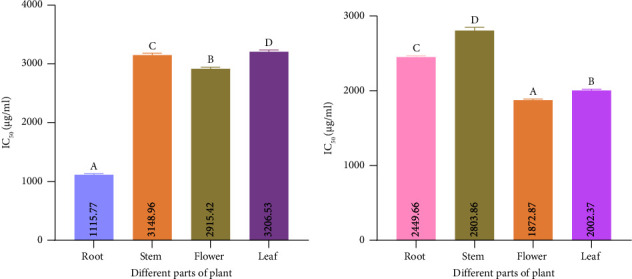
In vitro antioxidant activity of different solvent extracts of different parts in *Catharanthus roseus*. (a) Free radical scavenging capacity of ethanol extract in ABTS assay. (b) Free radical scavenging capacity of mixed-solvent extract in ABTS assay. Data are shown as mean with standard deviation for triplicate experiments. We employed the one-way ANOVA (⁣^∗∗∗^*p* < 0.001) to compare multiple groups; we used the pairwise Tukey's Honest Significant Difference (HSD) test to compare between two groups. In each group, the statistically significant (⁣^∗^*p* < 0.05) values are shown by superscripts A, B, C, and D. A similar letter suggests no statistically significant difference between groups, and a dissimilar letter suggests a statistically significant difference between groups. For the ethanol extract in the ABTS assay, Tukey's multiple comparisons test showed the following results: ⁣^∗^*p* < 0.05 for stem versus leaf, and ⁣^∗∗∗^*p* < 0.001 for flower versus leaf, root versus stem, root versus flower, root versus leaf, and stem versus flower. For the mixed-solvent extract in the ABTS assay, Tukey's multiple comparisons test revealed the following: ⁣^∗∗∗^*p* < 0.001 for flower versus leaf, root versus stem, root versus flower, root versus leaf, stem versus leaf, and stem versus flower.

The result demonstrated that the ABTS scavenging activity of four samples of two types of extract was significantly different from each other.  Figure 7(b) shows that among the four samples of mixed-solvent extract, the minimum value of IC_50_ was shown by the flower about 1872.87 *μ*g/mL and the maximum value was 2803.46 *μ*g/mL in the stem. In the case of ethanol extract, it ranged from a maximum of 3206.53 *μ*g/mL in leaf to a minimum of 1115.77 *μ*g/mL in root (Figure 7(a)).

#### 3.6.2. DPPH Scavenging Activity

**Figure 8:**
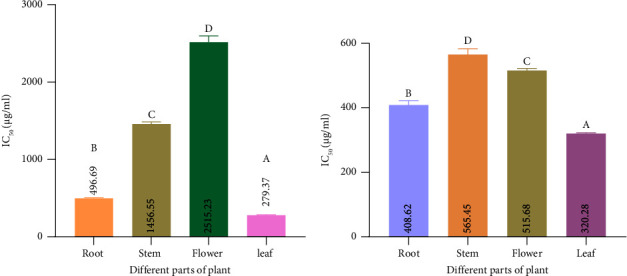
In vitro antioxidant activity of different solvent extracts of different parts in *Catharanthus roseus*. (a) Free radical scavenging capacity of ethanol extract in DPPH assay. (b) Free radical scavenging capacity of mixed-solvent extract in DPPH assay. Data are shown as mean with standard deviation for triplicate experiments. We employed the one-way ANOVA (⁣^∗∗∗^*p* < 0.001) to compare multiple groups; we used the pairwise Tukey's Honest Significant Difference (HSD) test to compare between two groups. In each group, the statistically significant (⁣^∗^*p* < 0.05) values are shown by superscripts A, B, C, and D. A similar letter suggests no statistically significant difference between groups, and a dissimilar letter suggests a statistically significant difference between groups. For the ethanol extract in the DPPH assay, Tukey's multiple comparisons test showed the following results: ⁣^∗∗∗^*p* < 0.001 for flower versus leaf, root versus stem, root versus flower, root versus leaf, stem versus leaf, and stem versus flower. For the mixed-solvent extract in the DPPH assay, Tukey's multiple comparisons test revealed the following: ⁣^∗∗∗^*p* < 0.001 for flower versus leaf, root versus stem, root versus flower, root versus leaf, stem versus leaf, and stem versus flower.

DPPH is one of the greatest antioxidant processes for determining the scavenging activity of radicals.  Figure 8(b) shows that among the four samples of mixed-solvent extract, the minimum value of IC_50_ was 320.28 *μ*g/mL shown by the leaf and the maximum value was 565.45 in the stem. In the case of ethanol extract, it ranged from a maximum of 2515.23 *μ*g/mL in flower to a minimum of 279.37 *μ*g/mL in leaf (Figure 8(a)). And each value of a different extract is identical to each other.

#### 3.6.3. Metal Chelating Capacity

**Figure 9:**
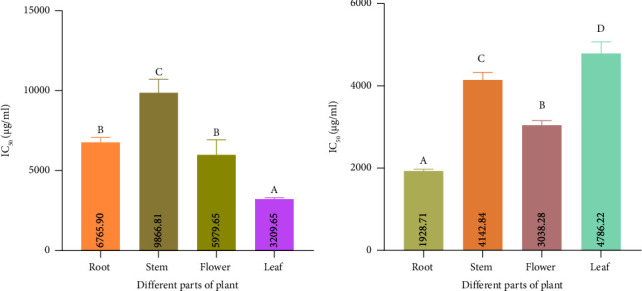
Metal chelating assay of different extracts. (a) Determination of IC_50_ value of metal chelating power for ethanol extract. (b) Determination of IC_50_ value of metal chelating power for mixed-solvent extract. Data are shown as mean with standard deviation for triplicate experiments. We employed the one-way ANOVA (⁣^∗∗∗^*p* < 0.001) to compare multiple groups; we used the pairwise Tukey's Honest Significant Difference (HSD) test to compare between two groups. In each group, the statistically significant (⁣^∗^*p* < 0.05) values are shown by superscripts A, B, C, and D. Similar letter suggests no statistically significant difference between groups, and dissimilar letter suggests the statistically significant difference between groups. For the ethanol extract in the metal chelating assay, Tukey's multiple comparisons test showed the following results: ⁣^∗∗∗^*p* < 0.001 for flower versus leaf, root versus stem, root versus leaf, stem versus leaf, and stem versus flower. Root versus flower group is not statistically significant (*p* > 0.05) for the ethanol extract in the metal chelating assay. For the mixed-solvent extract in the metal chelating assay, Tukey's multiple comparisons test revealed the following: ⁣^∗∗∗^*p* < 0.001 for flower versus leaf, root versus stem, root versus flower, root versus leaf, stem versus leaf, and stem versus flower.

The capacity to chelate metals is crucial because it lowers the metal concentration, which catalyzes the oxidation of lipids. In addition, because they lower the redox potential and hence stabilize the oxidized metal ions, metal-chelating compounds are regarded as secondary antioxidants. Root extract from mixed-solvent extract showed the minimum value of IC_50_ of 1928.71 *μ*g/mL, whereas the maximum value was 4786.22 in leaf (Figure 9(b)). In the case of ethanol extract, it ranged from a maximum of 9866.81 *μ*g/mL in the stem to a minimum of 3209.65 *μ*g/mL in leaf (Figure 9(a)).

#### 3.6.4. Reducing Power

**Figure 10:**
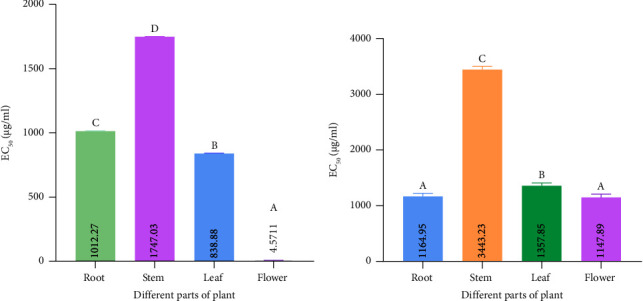
Reducing power assays of different extracts. (a) Determination of EC_50_ value for the power of ethanol extract and comparison among different samples. (b) Determination of EC_50_ value for reducing power of mixed-solvent extract and comparison among different samples. Data are shown as mean with standard deviation for triplicate experiments. We employed the one-way ANOVA (⁣^∗∗∗^*p* < 0.001) to compare multiple groups; we used the pairwise Tukey's Honest Significant Difference (HSD) test to compare between two groups. In each group, the statistically significant values are shown by superscripts A, B, C, and D. A similar letter suggests no statistically significant difference between groups, and a dissimilar letter suggests a statistically significant difference between groups. For the ethanol extract in the reducing power assay, Tukey's multiple comparisons test showed the following results: ⁣^∗∗∗^*p* < 0.001 for flower versus leaf, root versus stem, root versus flower, root versus leaf, stem versus leaf, and stem versus flower group. For the mixed-solvent extract in the reducing power assay, Tukey's multiple comparisons test revealed the following: ⁣^∗∗∗^*p* < 0.001 for root versus stem, stem versus leaf, and stem versus flower group. For the mixed solvent, Tukey's multiple comparisons test showed that root versus leaf (⁣^∗^*p* < 0.05) and flower versus leaf (⁣^∗∗^*p* < 0.01) groups are statistically significant. Root versus flower group is not statistically significant (*p* > 0.05) for the mixed-solvent extract in the metal chelating assay.

In the case of reducing power, ascorbic acid was used as the reference compound, and it indicates the transformation of ferric (III) from ferrous (II) form. The RP was identified to be higher with increasing concentration. Reducing power capacities of different samples' were represented by EC_50_. The term EC_50_ refers to the extract's half-maximum effective concentration (EC_50_) for reducing free radicals. Results showed (Figures 10(a) and 10(b)) that the EC_50_ of several extracts considerably differs. It was found that the EC_50_ value of root in ethanol extract was 1012.26 *μ*g/mL and that in mixed-solvent extract was 1164.947 *μ*g/mL. For the stem, the EC_50_ value in ethanol extract was 1747.03 *μ*g/mL, whereas in the mixed-solvent extract of the stem, it was 3443.23 *μ*g/mL. The findings also showed that the EC_50_ value of the flower in ethanol extract was 4.57114 *μ*g/mL and that in the mixed-solvent extract was 1147.89 *μ*g/mL. The EC_50_ value of the leaf in ethanol extract was 838.88 *μ*g/mL and that in the mixed-solvent extract was 1357.84 *μ*g/mL.

### 3.7. Molecular Docking Studies

**Figure 11:**
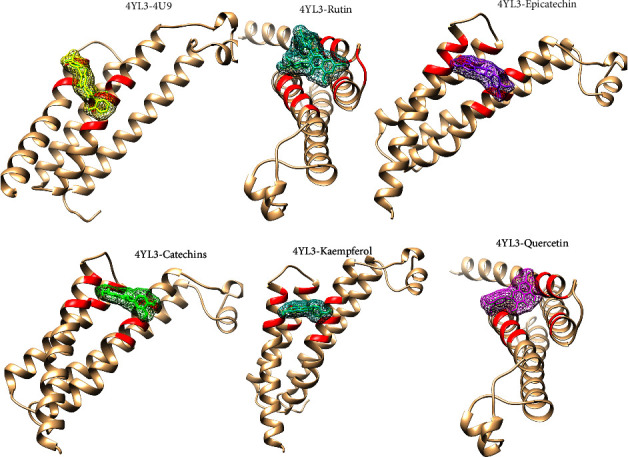
The 3-dimensional interaction of reference structure (5-[4-bromo-2-(2-chloro-6-fluorophenyl)-1H-imidazol-5-yl]-2-{[4-(trifluoromethyl) phenyl]ethynyl}pyridine) and HPLC-based quantified compounds to the ligand binding site of mPGES-1.

**Figure 12:**
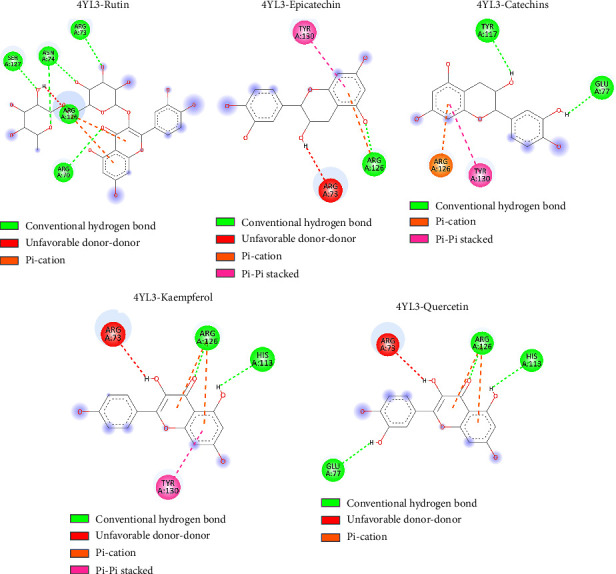
The 2-dimensional interaction of HPLC-based quantified compounds to the ligand binding site of mPGES-1.

Prostaglandin H2 (PGH2) is converted to prostaglandin E2 (PGE2) by an enzyme mPGES-1. Since mPGES-1 generated ROS, one potential therapeutic approach for the management of pain, inflammation, and some malignancies is the inhibition of mPGES-1 [29, 30]. We revealed that the quantified phytochemicals interact with the mPGES-1 with appreciable binding affinity (Table 3). The ranges of binding affinity were −4.5 kcal/mol to −7.1 kcal/mol (Table 3). Rutin, quercetin, epicatechin, catechins, and kaempferol interact with mPGES-1 with binding affinity lower than −6.0 kcal/mol (Table 3 and Figure 11). Moreover, we re-docked the reference co-crystal inhibitor 5-[4-bromo-2-(2-chloro-6-fluorophenyl)-1H-imidazol-5-yl]-2-{[4-(trifluoromethyl)phenyl]ethynyl}pyridine (4U9) with mPGES-1 to compare the binding affinity with our selected ligands. We found that 4U9 interacts with the protein with a binding affinity of −6.8 kcal/mol in the respective ligand binding site (Table 3). We revealed that rutin interacts with ARG A: 70, ARG A: 73, ASN A: 74, ARG A: 126, and SER A: 127 amino acid residues of mPGES-1 via four hydrogen bonds (Figure 12) and other chemical bonds. Similarly, the other phytocompounds, including quercetin, epicatechin, catechins, and kaempferol interact with the amino acid residues of mPGES-1 (Figure 12). Moreover, epicatechin interacts with three amino acid residues (ARG A: 73, TYR A: 130, and ARG A: 126), catechins interact with four amino acid residues (GLU A: 77, TYR A: 117, TYR A: 130, and ARG A: 126), kaempferol interacts with four amino acid residues (ARG A: 73, HIS A: 113, TYR A: 130, and ARG A: 126), and quercetin interacts with four amino acid residues (ARG A: 73, HIS A: 113, GLU A: 77, and ARG A: 126) in the ligand binding site of mPGES-1 (Figure 12). Among the quantified phytocompounds, rutin, quercetin, epicatechin, catechins, and kaempferol showed the lowest docking score (Table 3), indicating that these compounds are potent inhibitors of mPGES-1 for reducing the generation of ROS.

## 4. Discussion

*Catharanthus roseus* has a long history of traditional use in treating various ailments, including diabetes, malaria, cancer, and Hodgkin's lymphoma. Researchers found numerous alkaloids in *Catharanthus roseus* including vinblastine, vindoliscine, vincristine, vindensine, ajmalicine, reserpine, vincoline, vinacardine, catharanthamine, and vincardine. Vincristine and vinblastine were discovered to have powerful anticancer capabilities, and they were later developed into chemotherapeutic medications for the treatment of leukemia and other cancers [31]. *Catharanthus roseus* is enriched with alkaloids, flavonoids, carbohydrates, triterpenoids, quinone, tannins, saponins, and phenolic compounds. The experiment on *Catharanthus roseus* revealed the existence of bioactive substances that are significant for both biological and therapeutic purposes. In the study, various solvents were used to analyze the phytochemically active constituents subject to the plant. During the identification procedure, it was discovered that different solvent extracts had varying concentrations of phytochemicals.

Proximate analysis was conducted for estimation of the quantitative of food and food substance of different parts of *Catharanthus roseus* such as root, stem, flower, and leaf. In our study, ash contents were evaluated within a range of a minimum of 5.50% in the stem to a maximum value of 11.88% in the leaf. A study revealed that the ash content for the leaf was 15.21% and that for the flower was 8.37% which is relatable with our findings [23]. Moreover, the ash content in the stem was 4.76. which is relevant to our findings [32]. It has been found that the leaf contains higher protein about 13.62% than other parts of the plant. According to a study, the leaf contained higher protein than other parts of the plant which justifies our findings. The protein content of leaves was 8.08% higher than other parts [23]. The finding was that the leaf contained higher energy about 326.54 (⁣^∗∗∗^*p* < 0.001) than other parts of the plant. A study demonstrated that the leaf had a caloric value of 369.37 kcal which is close to our findings [33]. According to the findings of the current study, *Catharanthus roseus* implies high values for minerals together with a comparison of all analyzed samples and showed significant differences (⁣^∗∗^*p* < 0.001) among the individual mineral concentrations of each sample. The most significant discovery of the study was that *Catharanthus roseus* leaves had higher concentrations of all minerals except for Fe and Zn [34]. In the stem, potassium and calcium content was found about 106.0 and 159.3 g/100 g, respectively. Magnesium content was found in the root (162.4) as the highest [35].

We employed two distinct solvent systems for our extractions: first one is using ethanol alone and the other employing a combination of chloroform, methanol, and water (mixed solvents). The choice to use the mixed solvents was based on their ability to produce a significantly higher yield of extracts, as documented in previous studies [19]. Our results showed that using the mixed solvents yielded the following percentages from different plant parts: root (12.72%), stem (13.08%), leaf (36.79%), and flower (54.26%). In contrast, the extraction with ethanol alone resulted in the following yield: root (8.11%), stem (8.93%), leaf (40.05%), and flower (38.08%). Overall, our findings are in line with those of previous research, confirming the effectiveness of mixed solvents in enhancing the yield of certain plant extracts. However, the yield of the extract from the leaf was higher with ethanol alone compared to the mixed solvents. The outcomes of the experiment revealed that the mixed-solvent *extract* of *Catharanthus roseus* contained an especially high concentration of catechin hydrate which is found in the leaf sample. The experimental findings also demonstrated that the leaf was plentiful in trans-ferulic acid, quercetin, catechol, and (−) epicatechin. Rutin hydrate was higher in the root of the mixed-solvent extract. Vanillic acid and kaempferol were higher in flowers in comparison to other parts of the mixed-solvent extract. The mixed-solvent extract showed a higher value of catechin hydrate, rutin hydrate, trans-ferulic acid, and vanillic acid than ethanol extract. On the other hand (−) epicatechin, catechol, kaempferol, quercetin, and trans-cinnamic acid were in higher amounts in ethanol extract. Catechol, (−), epicatechin, rutin hydrate, and kaempferol were found higher, respectively, in root, stem, leaf, and flower in ethanol extract. Not only rutin hydrate but also catechin hydrate, trans-ferulic acid, quercetin, and trans-cinnamic acid were found in higher amounts in the leaf of the ethanol extract of *Catharanthus roseus*.

Our study observed significant differences in the TPC between extracts, ethanol and mixed solvent. Another investigation assessed the antioxidant activity of different parts of the *Catharanthus roseus* plant using the DPPH assay and TPC. The findings revealed that among the plant parts, the flower petals exhibited the highest total phenolic concentration, followed by the base and stem, while the leaves showed the lowest phenolic content. This information aligns with the study's conclusion that *Catharanthus roseus* demonstrates a complementary antioxidant effect [36]. The total amount of phenols found in methanol extract was (29.1 ± 0.8%) with the highest phenolic content followed by hot aqueous extract (22.5 ± 0.9%) [37]. Our study revealed that ethanol extract showed a good amount of total flavonoids except flower in comparison to mixed-solvent extract. Total flavonoid content was highest in the methanol extract (31.1) followed by the acetone extract (17.7) [37]. For total antioxidant analysis by phosphomolybdenum blue and FRAP assay, ethanol showed significantly high antioxidant properties in comparison to mixed-solvent extract. The average IC_50_ value for ethanol extract of root was 496.68 which was higher than the average IC_50_ value of mixed-solvent extract of root (408.62 *μ*g/mL). A study found the maximum DPPH radical scavenging activity of root extract of *Catharanthus roseus* as 80.68 ± 0.96% at 120 *μ*g/mL concentrations. The IC_50_ was found to be 57.39 *μ*g/mL [38].

In subject to ABTS, DPPH, and metal chelating assay, interestingly, mixed-solvent extract showed a lower IC_50_ value than ethanol extract when compared to four parts of the plant. IC_50_ of the metal chelating assay was lowest in root about 1928.71 *μ*g/mL. Though the flower, leaf, and stem of the mixed-solvent extract showed lower IC_50_ for ABTS assay, the root of ethanol extract showed the lowest IC_50_ (1115.77 *μ*g/mL). Also, for DPPH, the ethanol extract of the leaf showed the lowest IC_50_ (279.37 *μ*g/mL). Though total antioxidant properties and total flavonoid content were high in ethanol extract, the mixed-solvent extract showed higher EC_50_ for reducing power capacity and lower IC_50_ for ABTS, DPPH, and metal chelating ability. The EC_50_ value of the root in ethanol extract was 1012.268 and that in mixed-solvent extract was 1164.947. Another study revealed with an EC_50_ value of 57.79 g/mL, the maximum Fe^3+^ reduction was determined to be 76.28 0.41% at 120 g/mL concentration. The significant antioxidant potential of the root extract is evident from its notable reducing power [38]. Our findings deviate slightly from those of other researchers, and this discrepancy can be attributed to the influence of various factors. The variation in phytocompound content is dependent on several key factors such as geographical conditions, the plant's growth environment, climate, weather patterns, and other influencing variables [39, 40]. These divergent elements introduce a level of complexity to our study, emphasizing the importance of considering a multitude of environmental factors when interpreting and comparing results across different research studies. In addition, the antioxidant capacities and compound compositions differ among extracts obtained from different solvents and the number of extraction steps [41]. The choice of solvent combination significantly influences the extraction of antioxidants. Furthermore, there is a notable distinction between extracts obtained using organic solvent-water mixtures and those obtained with pure organic solvents [42]. The biological activity of an extract is markedly influenced by the extraction solvents employed, as they play a pivotal role in determining both the extraction yield and the content of bioactive compounds. This dual impact underscores the intricate relationship between solvent selection and the ultimate efficacy of the extracted material in various biological applications [21, 43]. In the end, the choice of extraction solvents is a critical determinant of the biological activity of an extract. A comprehensive understanding of how solvents influence extraction yield and bioactive compound content is essential for researchers seeking to harness the full therapeutic potential of natural products.

mPGES-1 is an enzyme that changes PGH2 into PGE2 [29, 30]. PGE2 induces the production of ROS which ultimately increases immune cell–mediated tumor growth and invasion, reduces apoptosis, increases metastasis and angiogenesis, and suppresses antitumor immunity [44]. mPGES-1-mediated PGE2 production contributes to inflammation, which is closely linked to ROS generation. ROS are reactive molecules that can cause cellular damage, contributing to chronic inflammation and associated diseases, including cardiovascular diseases, cancer, and neurodegenerative disorders [29, 30]. ROS can cause damage to lipids, proteins, and DNA, leading to cellular dysfunction and contributing to disease progression [45]. The link between mPGES-1 activity and ROS generation underscores the importance of targeting this enzyme to mitigate oxidative stress. We revealed that the quantified phytochemicals are interacting with mPGES-1 in the ligand binding site (Figures 11 and 12). Altogether, mPGES-1 could be potentially inhibited by rutin, quercetin, epicatechin, catechins, and kaempferol. Further experimental and clinical validation would be warranted to apply these findings in the clinical field.

## 5. Conclusion

*Catharanthus roseus* is a valuable and adaptable plant with a variety of applications in contemporary pharmacology and traditional medicine. Overall, *Catharanthus roseus* continues to be a significant and fascinating research topic for both scientists and plant enthusiasts due to its significant bioactive compounds with numerous pharmacological qualities. According to the findings of the current study, *Catharanthus roseus* implies a significant amount of minerals and antioxidant properties. In the ethanol extract from the root, we identified catechin hydrate, catechol, and (−) epicatechin. The stem ethanol extract contained catechin hydrate, vanillic acid, trans-ferulic acid, quercetin, trans-cinnamic acid, and kaempferol. For the flower ethanol extract, we found catechin hydrate, (−) epicatechin, trans-ferulic acid, quercetin, trans-cinnamic acid, and kaempferol. In the leaf ethanol extract, the compounds present were catechin hydrate, (−) epicatechin, rutin hydrate, trans-ferulic acid, quercetin, and trans-cinnamic acid. In the mixed-solvent extract from the root, we identified catechin hydrate, catechol, (−) epicatechin, rutin hydrate, and trans-ferulic acid. The mixed-solvent extract from the stem contained catechin hydrate, (−) epicatechin, vanillic acid, rutin hydrate, trans-ferulic acid, and quercetin. For the flower mixed-solvent extract, we found catechin hydrate, vanillic acid, rutin hydrate, and kaempferol. The mixed-solvent extract from the leaf included catechin hydrate, (−) epicatechin, vanillic acid, trans-ferulic acid, and quercetin. Our findings revealed that several compounds from this plant extracts, such as rutin, quercetin, epicatechin, catechins, and kaempferol, interact with the ligand binding site of mPGES-1, effectively inhibiting the enzyme. Altogether, our study revealed a dual action of *Catharanthus roseus*, where the extracts displayed notable antioxidant capacity, highlighting their potential in combating oxidative stress. Simultaneously, the plant's phytoconstituents exhibited significant inhibitory activity against the ROS-generating enzyme (mPGES-1). The presence of this dual functionality accentuates the comprehensive antioxidant profile, exploring effectiveness in neutralizing a wide array of free radicals. The identification of specific phytocompounds responsible for these effects opens promising avenues for further research and potential applications in developing novel chemotherapeutic agents aimed at inhibiting and eliminating ROS. These findings contribute valuable insights to the field of natural compounds with therapeutic potential against oxidative stress–related conditions. Based on findings, future research should focus on several key areas to further exploit the potential of *Catharanthus roseus*. First, conduct in vivo studies to assess the effectiveness of its extracts and compounds in reducing oxidative stress and inflammation. Second, develop novel pharmaceutical formulations or delivery systems to enhance the efficacy and stability of its bioactive compounds. Third, explore potential synergistic effects between different phytocompounds in the plant. Fourth, establish sustainable cultivation practices to ensure a reliable supply of high-quality material. Finally, evaluate the public health impact of *Catharanthus roseus*–based interventions, including their cost-effectiveness and accessibility for managing oxidative stress–related conditions.

## Figures and Tables

**Table 1:** Proximate analysis of different parts of *Catharanthus roseus.*

**Sample**	**Moisture (g/100 g)**	**Ash (g/100 g)**	**Protein (g/100 g)**	**Fat (g/100 g)**	**Carbohydrate (g/100 g)**	**Fiber (g/100 g)**	**Energy (Kcal/100 g)**
Root	2.87 ± 0.15^a^	5.89 ± 0.07^b^	6.39 ± 0.12^b^	1.23 ± 0.14^a^	38.08 ± 0.13^b^	45.69 ± 0.05^b^	188.91 ± 0.36^b^
Stem	4.016 ± 0.15^b^	5.50 ± 0.10^a^	5.47 ± 0.15^a^	4.41 ± 0.18^b^	26.57 ± 0.13^a^	54.18 ± 0.09^c^	168.03 ± 0.23^a^
Flower	10.14 ± 0.16^c^	7.76 ± 0.07^c^	7.90 ± 0.06^c^	4.27 ± 0.07^b^	60.37 ± 0.06^d^	9.68 ± 0.06^a^	311.44 ± 0.26^c^
Leaf	4.102 ± 0.21^b^	11.88 ± 0.07^d^	13.62 ± 0.11^d^	6.05 ± 0.15^c^	54.88 ± 0.89^c^	9.81 ± 0.04^a^	326.54 ± 0.27^d^

Data are shown as mean with standard deviation for triplicate experiments. We employed the one-way ANOVA to compare multiple groups; we used the pairwise Tukey's Honest Significant Difference (HSD) test to compare between two groups. In each group, the statistically significant (⁣^∗^*p* < 0.05) values are shown by superscripts a, b, c, and d. A similar letter suggests no statistically significant difference between groups, and a dissimilar letter suggests a statistically significant difference between groups.

**Table 2:** The mineral content (mg/kg) of *Catharanthus roseus.*

**Sample**	**Sodium**	**Potassium**	**Calcium**	**Magnesium**	**Iron**	**Manganese**	**Zinc**
Root	915.28 ± 16.39^c^	5614 ± 42.88^c^	2706.26 ± 63.15^b^	1286.62 ± 39.92^a^	8553.78 ± 38.82^c^	2759.56 ± 87.14^b^	8453.6 ± 60.75^a^
Stem	1225.86 ± 62.34^d^	9836.52 ± 96.73^d^	5285.18 ± 83.25^c^	1641.6 ± 69.90^b^	6699.38 ± 91.20^a^	6571.62 ± 22.78^d^	8458.4 ± 35.07^a^
Flower	382.9 ± 14.18^a^	1138.82 ± 24.52^a^	7171.3 ± 67.12^d^	1972.68 ± 18.84^c^	8056.22 ± 20.05^b^	2575.18 ± 24.85^a^	12,321.04 ± 99.70^b^
Leaf	818.46 ± 25.80^b^	1860.24 ± 24.77^b^	2359.26 ± 31.55^a^	4441.52 ± 21.78^d^	9757.06 ± 28.57^d^	6363.14 ± 64.36^c^	8546.36 ± 25.71^a^

Data are shown as mean with standard deviation for triplicate experiments. We employed the one-way ANOVA to compare multiple groups; we used the pairwise Tukey's Honest Significant Difference (HSD) test to compare between two groups. In each group, the statistically significant (⁣^∗^*p* < 0.05) values are shown by superscripts a, b, c, and d. A similar letter suggests no statistically significant difference between groups, and a dissimilar letter suggests a statistically significant difference between groups.

**Table 3:** The binding affinity of the quantified phytochemicals and reference compound (4U9: 5-[4-bromo-2-(2-chloro-6-fluorophenyl)-1H-imidazol-5-yl]-2-{[4-(trifluoromethyl)phenyl]ethynyl}pyridine).

**Phytocompounds**	**Binding affinity (Kcal/mol)**	**rmsd/ub**	**rmsd/lb**
Rutin	−7.1	0	0
4U9	−6.8	0	0
Quercetin	−6.6	0	0
Epicatechin	−6.3	0	0
Catechins	−6.3	0	0
Kaempferol	−6.1	0	0
trans-Ferulic acid	−5.4	0	0
Vanillic acid	−5	0	0
Cinnamic acid	−5	0	0
Catechol	−4.5	0	0

## Data Availability

The protein structure was downloaded from the RCSB Protein Data Bank (https://www.rcsb.org/). The SMILE structure of all selected compounds was downloaded from PubChem (https://pubchem.ncbi.nlm.nih.gov/).
